# Massage

**Published:** 1888-02

**Authors:** 


MASSAGE: PRINCIPLES AND PRACTICE OF REMEDIAL TREATMENT BY IMPARTED MOTION. Mechanical Processes. By Geo. H. Taylor, M. D. New York: John B. Alden, 1887.

This book is interesting as far as its facts go (and that is not far), but very stupid when the author begins to theorize. It aims to teach one to cure himself—a very foolish attempt, for it leaves no room for quackery and thus knocks out the author in the first round. H. H.

